# Preparation and Characterization of Electrospun Fluoro-Containing Poly(imide-benzoxazole) Nano-Fibrous Membranes with Low Dielectric Constants and High Thermal Stability

**DOI:** 10.3390/nano11020537

**Published:** 2021-02-19

**Authors:** Meng-Ge Huangfu, Deng-Xiong Shen, Xin-Xin Zhi, Yan Zhang, Yan-Jiang Jia, Yuan-Cheng An, Xin-Ying Wei, Jin-Gang Liu

**Affiliations:** 1 School of Materials Science and Technology, China University of Geosciences, Beijing 100083, China; 2103180029@cugb.edu.cn (M.-G.H.); 3003200015@cugb.edu.cn (X.-X.Z.); 3003200016@cugb.edu.cn (Y.Z.); 2003190024@cugb.edu.cn (Y.-J.J.); 2103190039@cugb.edu.cn (Y.-C.A.); 2003200022@cugb.edu.cn (X.-Y.W.); 2 Aerospace Research Institute of Materials& Processing Technology, Beijing 100076, China; shendx86@gmail.com

**Keywords:** polyimide, polybenzoxazole, nano-fibrous membrane, electrospinning, dielectric properties

## Abstract

The rapid development of advanced high-frequency mobile communication techniques has advanced urgent requirements for polymer materials with high-temperature resistance and good dielectric properties, including low dielectric constants (low-*D*_k_) and low dielectric dissipation factors (low-*D*_f_). The relatively poor dielectric properties of common polymer candidates, such as standard polyimides (PIs) greatly limited their application in high-frequency areas. In the current work, benzoxazole units were successfully incorporated into the molecular structures of the fluoro-containing PIs to afford the poly(imide-benzoxazole) (PIBO) nano-fibrous membranes (NFMs) via electrospinning fabrication. First, the PI NFMs were prepared by the electrospinning procedure from organo-soluble PI resins derived from 2,2′-bis(3,4-dicarboxy-phenyl)hexafluoropropane dianhydride (6FDA) and aromatic diamines containing *ortho*-hydroxy-substituted benzamide units, including 2,2-bis[3-(4-aminobenzamide)-4-hydroxylphenyl]hexafluoropropane (*p*6FAHP) and 2,2-bis[3-(3-aminobenzamide)-4-hydroxyphenyl]hexafluoropropane (*m*6FAHP). Then, the PI NFMs were thermally dehydrated at 350 °C in nitrogen to afford the PIBO NFMs. The average fiber diameters (*d*_av_) for the PIBO NFMs were 1225 nm for PIBO-1 derived from PI-1 (6FDA-*p*6FAHP) precursor and 816 nm for PIBO-2 derived from PI-2 (6FDA-*m*6FAHP). The derived PIBO NFMs showed good thermal stability with the glass transition temperatures (*T*_g_s) over 310 °C and the 5% weight loss temperatures (*T*_5%_) higher than 500 °C in nitrogen. The PIBO NFMs showed low dielectric features with the *D*_k_ value of 1.64 for PIBO-1 and 1.82 for PIBO-2 at the frequency of 1 MHz, respectively. The *D*_f_ values were in the range of 0.010~0.018 for the PIBO NFMs.

## 1. Introduction

The rapid development of telecommunication techniques has witnessed the start of fifth-generation (5G) era in recent years [1]. One of the most extraordinary features of 5G communication is the high-frequency transmission of signals in hardware [2]. This characteristic makes it very important to achieve low-loss and low-delay signals transmission [3]. However, the signal delay caused by various components in 5G hardware seems to be more serious with increasing frequency. It has been well established that the signal delay is closed with the dielectric constants of the materials that the signals penetrate [4,5,6]. The lower the dielectric constant (*D*_k_) and dielectric dissipation factor (*D*_f_) of the hardware dielectric materials, the smaller the delay of the signal transmission [7]. Thus, hardware dielectric materials with low-*D*_k_ and low-*D*_f_ characteristics are in demand for the 5G combination construction. Another important developing trend for research and development of 5G telecommunication is the combination with advanced display techniques, such as wearable display, health monitoring display, and so on [8]. Thus, among the new materials developed for 5G applications, advanced fabrics or fibrous membranes with low-*D*_k_ and low-*D*_f_ features have occupied important positions [9]. 

**Figure 1:**
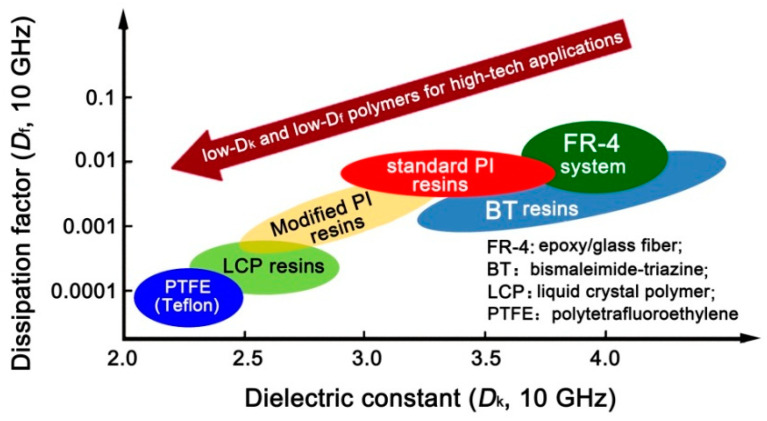
Common low-*D*_k_ and low-*D*_f_ polymers reported in the literature.

Polyimide (PI) has been widely used in microelectronic fields for many decades due to its good combined properties, including excellent thermal and environmental stability, good dielectric properties, and flexible molecular structure designability [10,11,12,13,14]. Conventional PIs, such as poly(pyromellitic dianhydride-4,4′-oxydianiline) (PI_PMDA-ODA_) usually possess *D*_k_ and *D*_f_ values around 3.2–3.5 and 0.01–0.10, respectively, at the dry state [15]. In order to decrease the *D*_k_ and *D*_f_ values of common PIs, various methodologies have been developed in the literature, including the introduction of substituents with low electronegativity and bulky molecular volume [16,17], or incorporation of low-*D*_k_ components, such as air, and so on [18,19,20]. However, the level of *D*_k_ and *D*_f_ values (2.0–2.5@1 MHz) obtained by the former modifications usually cannot meet the requirements of high-frequency signal transmission applications. For example, according to the prediction of IMAPS (International Microelectronics Assembly and Packaging Society), the *D*_k_ values for advanced polymeric dielectrics should be lower than 2.0 at the frequency of 1 MHz in 2019 [21]. For the latter modification, the mechanical properties of the derived air-filled PI systems are usually severely deteriorated. On the other hand, from the viewpoint of effects of molecular structure on the dielectric parameters of the polymers, the existence of polar and hydrophilic imide rings in PIs is prone to increasing the *D*_k_ and *D*_f_ values, especially during the servicing life in moist environments. Thus, high-temperature resistant polymers with intrinsic low-*D*_k_ and low-*D*_f_ characteristics, such as polybenzoxazole (PBO) [22], liquid crystal polymer (LCP), polytetrafluoroethylene (PTFE), and so on have attracted increasing attention in 5G new dielectric materials (Figure 1). Actually, low-*D*_k_ PBOs have been widely investigated as high-performance interlayer dielectrics for microelectronic fabrication in past decades [23,24,25]. However, few works have reported on the electrospun low-*D*_k_ PBO fibrous membranes up to now [26,27,28].

In the current work, as part of our continuous work developing high-performance polymers for microelectronic applications, a series of electrospun nano-fibrous membranes (NFMs) containing fluoro-substituted poly(imide-benzoxazole)s (PIBOs) were developed. The effects of various substituents, including hexafluoroisopropylidene, imide ring, and benzoxazole rings on the thermal, optical, and dielectric properties were investigated in detail.

## 2. Materials and Methods 

### 2.1. Materials

*p*-Nitrobenzoyl chloride (pNBC, purity: 98.0%) and *m*-nitrobenzoyl chloride (mNBC, purity: 98.0%) were purchased from Sigma-Aldrich (Shanghai, China) and used as received. 2,2-Bis(3-amino-4-hydroxyphenyl)hexafluoropropane (6FAP, purity: 99.5%) was purchased from Changzhou Sunlight Pharmaceutical Co., Ltd., (Jiangsu, China) and used as received. 2,2′-Bis(3,4-dicarboxyphenyl)hexafluoropropane dianhydride (6FDA, purity: >99.0%) was purchased from Tokyo Chemical Industry (TCI, Tokyo, Japan) and dried in vacuo at 180 °C for 12 h prior to use. The analytical grade of *N*-methyl-2-pyrrolidinone (NMP), *N*,*N*-dimethylacetamide (DMAc), and *N*,*N*-dimethylforamide (DMF) were distilled prior to use and stored under 4 Å molecular sieve. The other commercially available analytical grade of reagents including *m*-cresol, isoquinoline, absolute ethanol, pyridine, and palladium on active carbon (Pd/C) (5%) (Sinopharm Chemical Reagent Co. Ltd., Shanghai, China) were used as received.

### 2.2. Measurements

Inherent viscosity was measured using an Ubbelohde viscometer with a 0.5 g dL^−^^1^ NMP solution at 25 °C. The attenuated total reflectance Fourier transform infrared (ATR-FTIR) spectrum was obtained on a Bruker Tensor-27 FT-IR spectrometer (Ettlingen, Germany). Nuclear magnetic resonances (^1^H-NMR) were performed on an AV 400 spectrometer (Tokyo, Japan) operating at 400 MHz in deuterated dimethyl sulfoxide (DMSO-*d_6_)*. The number average molecular weight (*M*_n_) and weight average molecular weight (*M*_w_) of the PI resins were measured using a gel permeation chromatography (GPC) system (Shimadzu, Kyoto, Japan). Field-emission scanning electron microscopy (FE-SEM) was carried out using a Technex Lab Tiny-SEM 1540 (Tokyo, Japan) with an accelerating voltage of 15 KV for imaging. Pt/Pd was spattered on each film in advance of the measurements.

Ultraviolet-visible (UV-Vis) spectra were recorded on a Hitachi U-3210 spectrophotometer (Tokyo, Japan) at room temperature. Prior to test, PI fabric samples were dried at 100 °C for 10 h to remove the absorbed moisture. Whiteness of the PI nonwoven fabric was measured using an X-rite Ci7800 spectrophotometer (Grand Rapids, Michigan, MI, USA) in accordance with the procedure described in the Chinese standard GB/T 17644-2008 (Test method for whiteness and chromaticity of textile fibers) and in the standard of ISO 11475: 2017 (Paper and board- determination of CIE (Commission Internationale de l´Eclairage) whiteness, D65/10°, outdoor daylight). The color parameters were calculated according to a CIE Lab equation. L^*^ is the lightness, where 100 means white and 0 implies black. A positive a^*^ means a red color, and a negative one indicates a green color. A positive b^*^ means a yellow color, and a negative one indicates a blue color. The color parameters of a standard poly(pyromellitic dianhydride-oxydianiline) (PMDA-ODA) fabric was also measured for reference. The whiteness values of the PI mats were calculated as Equation (1).

*W* = 100 − [(100 − L*)^2^ + a*^2^ + b*^2^]^1/2^ (1)

where *W* standards for whiteness, L^*^ standards for index of lightness, a* and b* stand for chromaticity coefficient.

Thermogravimetric analysis (TGA) was performed on a TA-Q50 thermal analysis system (New Castle, Delaware, USA) at a heating rate of 20 °C min^−^^1^ in nitrogen. Differential scanning calorimetry (DSC) was carried on a TA-Q 100 thermal analysis system (New Castle, Delaware, USA) at a heating rate of 10 °C min^−^^1^ in nitrogen. 

Solubility was characterized as follows: 1.0 g of the PI resin was mixed into 9.0 g of the solvent tested (10 wt% solid content), which was stirred for 24 h at room temperature. The solubility was determined visually as three grades: completely soluble (++), partially soluble (+−), and insoluble (−), wherein complete soluble indicates a homogenous and clean state without phase separation, precipitation or gel formation, and insoluble indicates no change of the resin in the appearance.

The dielectric properties of PI and PBO NFMs were measured using an Agilent 4294 A precise impedance analyzer (Agilent Technologies Company, Palo Alto, CA, USA) at room temperature in the frequency range from 1000 Hz to 1 MHz. The samples were dried at 100 °C for 1 h to remove the absorbed moisture prior to test.

### 2.3. Monomer Synthesis

2,2-Bis [3-(4-aminobenzamide)-4-hydroxyphenyl]hexafluoropropane (*p*6FAHP) and 2,2-bis[3-(3-aminobenzamide)-4-hydroxyphenyl]hexafluoropropane (*m*6FAHP) were synthesized in our laboratory according to a modified route reported in the literature [29] except that 2,2-bis(3-amino-4-hydroxyphenyl)hexafluoropropane (6FAP) was used instead of 2,2-bis(4-hydroxy-phenyl)hexafluoropropane. The diamines obtained were purified by recrystallization from absolute ethanol for several times.

*p*6FAHP: yield: 76.4%. Purity (high-performance liquid chromatography, HPLC): 99.5%. Melting point: 298.4 °C (DSC peak temperature). ATR-FTIR (KBr, cm^−1^): 3439, 3364, 3144, 1639, 1605, 1537, 1508, 1422, 1381, 1321, 1254, 1190, 1146, and 1101. Nuclear magnetic resonance (^1^H-NMR) (400 MHz, DMSO-*d*_6_, ppm): 10.39 (s, 2H), 9.25 (s, 2H), 7.96 (s, 2H), 7.70–7.67 (d, 4H), 7.00–6.93 (m, 4H), 6.62–6.59 (d, 4H), and 5.84 (s, 4H). ^13^C-NMR (100 MHz, DMSO-*d*_6_, ppm): 166.1, 153.0, 149.4, 129.9, 127.1, 126.8, 126.0, 124.6, 123.7, 123.6, 121.5, 120.5, 116.5, 113.3, and 67.5. Elemental analysis: calculated for C_29_H_22_F_6_N_4_O_4_: C, 57.62%, H, 3.67%, N, 9.27%; found: C, 57.33%, H, 3.72%, N, 9.16%.

*m*6FAHP: yield: 74.3%. Purity (HPLC): 99.3%. Melting point: 318.8 °C (DSC peak temperature). ATR-FTIR (KBr, cm^−1^): 3410, 3371, 3148, 1649, 1584, 1547, 1514, 1435, 1362, 1331, 1300, 1256, 1192, 1138, and 1097. ^1^H-NMR (400 MHz, DMSO-*d*_6_, ppm): 10.44 (s, 2H), 9.27 (s, 2H), 8.07 (s, 2H), 7.18–7.10 (m, 6H), 7.07–7.02 (m, 4H), 6.79–6.75 (m, 2H), and 5.35 (s, 4H). ^13^C-NMR (100 MHz, DMSO-*d*_6_, ppm): 166.5, 149.4, 135.4, 129.6, 128.3, 127.1, 126.6, 126.0, 124.4, 123.7, 123.4, 121.4, 117.7, 116.0, 115.0, 113.1, and 67.5. Elemental analysis: calculated for C_29_H_22_F_6_N_4_O_4_: C, 57.62%, H, 3.67%, N, 9.27%; found: C, 57.41%, H, 3.75%, N, 9.23%.

### 2.4. Synthesis of Polyimide (PI) Resins

The PI resins were prepared from 6FDA and the *ortho*-hydroxyl-substituted benzamide- containing diamines via a one-step high-temperature polycondensation procedure, which could be illustrated by the synthesis of PI-1 (6FDA-*p*6FAHP). Into a 1000 mL four-necked flask reactor equipped with a mechanical stirrer (stainless steel covered with polytetrafluoroethylene), a heating equipment, a Dean-Stark trap, and a nitrogen inlet was charged with *p*6FAHP (12.0900 g, 20 mmol) and *m*-cresol (200.0 g) at 25 °C. Then, 6FDA (8.8848 g, 20 mmol) was added to the diamine solution, together with an additional *m*-cresol (41.2 g) to make the solid content of the reaction mixture to be 8 wt%. After stirring in nitrogen for 1 h, toluene (150 g) and isoquinoline (0.5 g) was added. The reaction mixture was then heated to 180 °C to perform the dehydrating reaction, during which the water by-products produced were removed via the toluene azeotrope at 140–145 °C for 6 h. Then, the toluene was distilled out of the reaction system until the inner temperature reached 180 °C. The imidization reaction was performed at 180 °C for 3 h, and then cooled to room temperature. The viscous solution obtained was precipitated into an excess of aqueous ethanol solution (80 vol%). The precipitated PI-1 resin was immersed into the aqueous ethanol for 24 h, and then collected. After drying at 100 °C in vacuo for 24 h, PI-1 resin was obtained as pale-yellow fibrous solid. Yield: 19.71 g (97.3%). ^1^H-NMR (DMSO-*d*_6_, ppm): 10.46 (s, 2H), 9.66 (s, 2H), 8.40–8.37 (m, 2H), 8.25–8.21 (m, 2H), 8.12–8.07 (m, 4H), 8.01–7.94 (m, 4H), 7.79–7.74 (m, 2H), 7.64–7.60 (m, 2H), and 7.13–7.00 (m, 4H).

PI-2 (6FDA-*m*6FAHP) resin was obtained by a similar procedure mentioned above except *p*6FAHP was changed to *m*6FAHP. Yield: 19.67 g (97.1%). ^1^H-NMR (DMSO-*d*_6_, ppm): 10.48 (s, 2H), 9.64 (s, 2H), 8.37–8.35 (m, 2H), 8.29–8.20 (m, 2H), 8.08–7.92 (m, 8H), 7.70–7.69 (m, 2H), and 7.12–7.00 (m, 4H).

### 2.5. Electrospinning Preparation of PI Nano-Fibrous Membranes (NFMs) and Poly(imide-benzoxazole) (PIBO) NFMs

PI-1 resin was dissolved in DMAc at room temperature with a solid content of 15 wt% to afford a solution. The PI-1 solution was purified by filtration through a 0.45 μm Teflon syringe filter. Then, the clear PI-1 solution was filled into a 5 mL syringe fabricated with a needle spinneret with an inner diameter of 0.21 mm. A syringe pump was used to squeeze out the PI-1 solution through the spinneret at the speed of 0.2 mL h^−^^1^. A positive voltage of 15 kV and a negative voltage of −3 kV were applied between the syringe and the collector. The distance between the spinneret and the grounded rotating drum collector (diameter: 10 cm; length: 30 cm) was 15 cm. The humidity in the electrospinning apparatus was controlled to be 50 ± 2% relative humidity. The rotating speed of the collector was set to be 100 rpm. The PI-1 fibers were deposited on the aluminum foil as the support medium on the collector. The obtained PI-1 NFM was dried at 120 °C in vacuo for 1 h to remove the residual solvent.

The PI-NFM with an aluminum carrier was put into a nitrogen-purged high-temperature clean oven and then thermally treated according to the procedure of 80 °C/1 h, 150 °C/1 h, 250 °C/1 h, and 350 °C/1 h. Then, the sample was cooled to room temperature. The PIBO NFM obtained was used for various measurements.

PI-2 and PIBO-2 NFM were prepared according to a similar procedure as mentioned above.

## 3. Results and Discussion

### 3.1. Monomoer Synthesis

**Figure 2:**
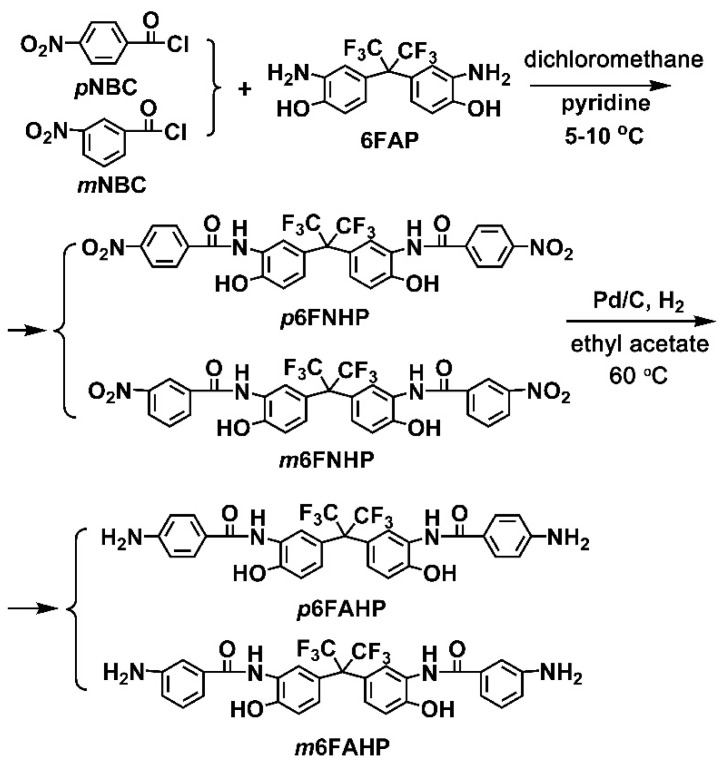
Synthesis of diamine monomers.

Two diamine monomers, p6FAHP and m6FAHP were synthesized by a two-step procedure shown in Figure 2. p-Nitrobenzoyl chloride (pNBC) or m-nitrobenzoyl chloride (mNBC) were first reacted with 6FAP at low temperature in the solvent to form the dinitro compounds. pyridine was used as the absorbent of the produced hydrogen chloride (HCl) by-products. HCl was removed from the reaction in the form of pyridine hydrochloride. In the reaction, the aromatic acyl chloride group (–COCl) was likely to react with both of the amine groups (–NH_2_) and hydroxyl (–OH) groups in the molecular structure of 6FAP. But at low reaction temperature, the acyl chloride is more likely to react with amino groups due to the relatively lower thermodynamic conditions required. At low temperature, the chance of producing by-products was effectively controlled. The dinitro compounds were then reduced by hydrogen under the catalysis of Pd/C to afford the target diamine monomers with high yields over 80%.

**Figure 3:**
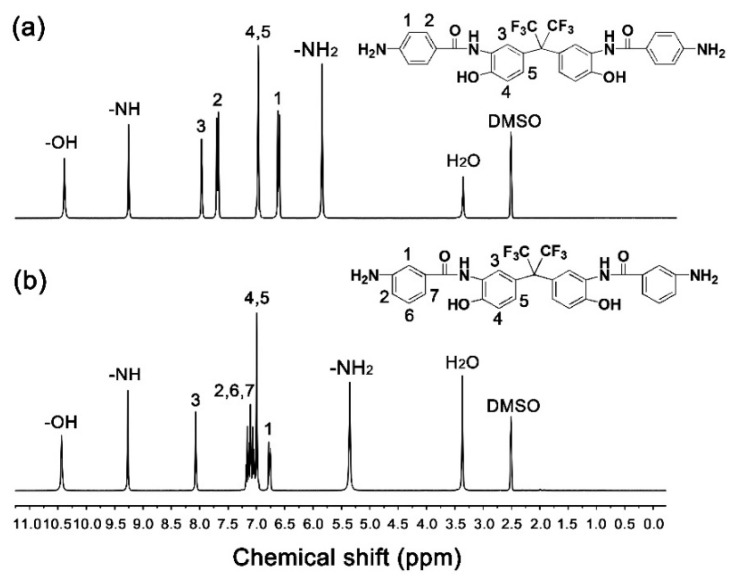
Nuclear magnetic resonance (^1^H-NMR) spectra of diamines (in deuterated dimethyl sulfoxide (DMSO-*d*_6_)). (**a**) 2,2-bis[3-(4-aminobenzamide)-4-hydroxylphenyl]hexafluoropropane (*p*6FAHP); (**b**) 2,2-bis[3-(3-aminobenzamide)-4-hydroxyphenyl]hexafluoropropane (*m*6FAHP).

The chemical structures of the diamine monomers were confirmed by various measurements as shown in Section 2.3. The typical ^1^H-NMR spectra of the diamines are shown in Figure 3. In both of the spectra, the absorption of protons on –OH appeared furthest downfield (*p*6FAHP: 10.39 ppm; m6FAHP: 10.44 ppm). The absorptions of protons in the –CONH– bonds were detected second furthest downfield in the spectra (*p*6FAHP: 9.25 ppm; *m*6FAHP: 9.27 ppm). The protons adjacent to the electron-withdrawing hexafluoroisopropylidene group (H_3_) showed the absorptions downfield in the spectra. By contrast, the protons in the amino group (–NH_2_) revealed the absorptions furthest upfield (*p*6FAHP: 5.84 ppm; *m*6FAHP: 5.35 ppm) except those of the solvents. This is consistent with the anticipated structures of the diamines.

### 3.2. PI Resins Synthesis and Electrospun PI NFMs Preparation

**Figure 4:**
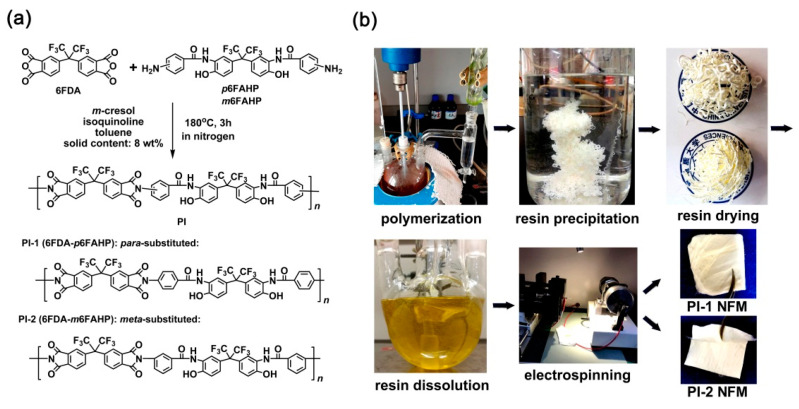
Synthesis of polyimide (PI) resins (**a**) and preparation of PI nano-fibrous membranes (NFMs) (**b**).

The fluoro-containing dianhydride, 6FDA was polymerized with fluoro-containing diamines, *p*6FAHP and *m*6FAHP, respectively to afford PI-1 (6FDA-*p*6FAHP) and PI-2 (6FDA-*m*6FAHP) by a one-step high-temperature polycondensation procedure, as shown in Figure 4a. The two aromatic diamines were synthesized and used to incorporate the *ortho*-hydroxyl-substituted benzamide units into the derived PIs, which was thought to be one of the most promising precursors for PBO synthesis. Generally, benzoxazole rings could be formed through several routes, such as the decarbonation (–CO_2_) and rearrangement reactions via *ortho*-hydroxyl substituted imide rings [30,31], the dehydration reaction via *ortho*-hydroxyl substituted benzamide [32], and so on. Compared with the former technical route of decarbonation, the latter dehydration route usually requires more mild thermodynamic and kinetic conditions to form benzoxazole. For example, the conversion temperature of the former usually exceed 450 °C, while the latter only needs 300–350 °C to achieve the complete conversion from the precursor to the benzoxazole.

PI resins were obtained as pale-yellow solids with good toughness, indicating moderate to high molecular weights. Table 1 shows the inherent viscosities ([*ƞ*]_inh_) and GPC results of the PI resins. The PI resins showed [*ƞ*]_inh_ value of 0.78 dL g^−1^ for PI-1 and 0.73 dL g^−1^ for PI-2, respectively, indicating the high polymerization reactivity of the diamine monomers. In addition, the PI resins showed numerical average molecular weights (*M*_n_) higher than 3.0 × 10^4^ g mol^−1^ and polydispersity index (PDI) values of 1.65 for PI-1 and 1.56 for PI-2, respectively. This also revealed the good reactivity of the monomers.

As shown in Table 1, the PI resins were soluble in common polar aprotic solvents (NMP, DMAc, DMF) at room temperature with a solid content higher than 10 wt%. The good solubility of the PI resins was mainly due to the existence of the bulky hexafluoroisopropyl groups both in the dianhydride and the diamine moieties, and also the presence of the –OH groups in the polymers. PI-2 was also soluble in chloroform, while PI-1 was partially soluble in the solvent. The better solubility of PI-2 was mainly attributed to the *meta*-substituted molecular structure in the diamine units. Both of the PI resins were insoluble in tetrahydrofuran (THF).

**Figure 5:**
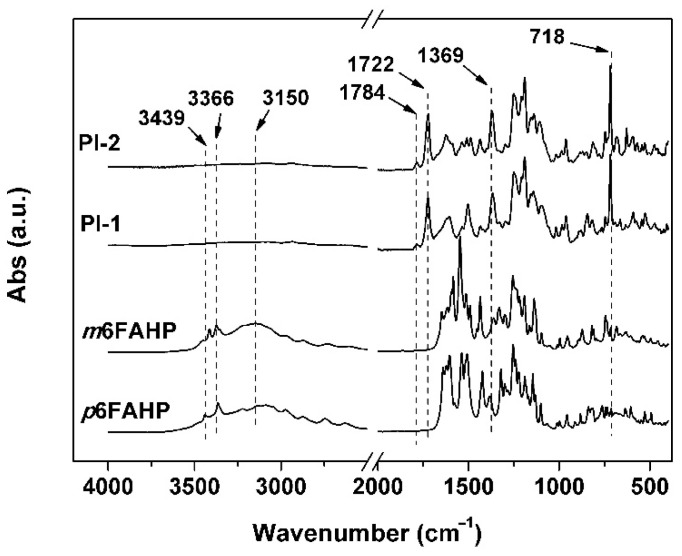
Attenuated total reflectance Fourier transform infrared (ATR-FTIR) spectra of diamines and derived PI nano-fibrous membranes (NFMs).

Good solubility of the PI resins in the organic solvents made it possible to fabricate the NFMs via a one-step electrospinning procedure, as shown in Figure 4b. PI-1 and PI-2 NFMs were successfully prepared using the common electrospinning conditions established in our previous work for the organo-soluble PI systems [33] and the 6FDA-based PI systems reported in the literatures [34,35,36,37]. The chemical structures of the PI NFMs were investigated by AFR-FTIR and ^1^H-NMR measurements, respectively. According to the AFR-FTIR spectra (Figure 5) of the PIs as well as the diamine monomers, the characteristic absorptions of the N–H stretching vibrations in the primary –NH_2_ of the diamines (3439 cm^−1^ and 3366 cm^−1^) totally disappeared in the spectra of the PIs. Alternatively, the characteristic absorptions of the imide rings, including the asymmetrical carbonyl stretching vibrations at the wavenumber of 1784 cm^−1^ and the symmetric ones at 1722 cm^−1^, the C–N stretching vibration at 1369 cm^−1^, and the bending vibrations of carbonyl at 718 cm^−1^ were all detected. This confirmed the full conversion from the monomers to the polymers. In addition, for both of the PI NFMs, the characteristic absorptions of C–F at 1190 cm^−1^, –O– at 1252 cm^−1^, and C=C at 1502 cm^−1^ were all recorded in the spectra. All the information proved the successful preparation of the PIs.

**Figure 6:**
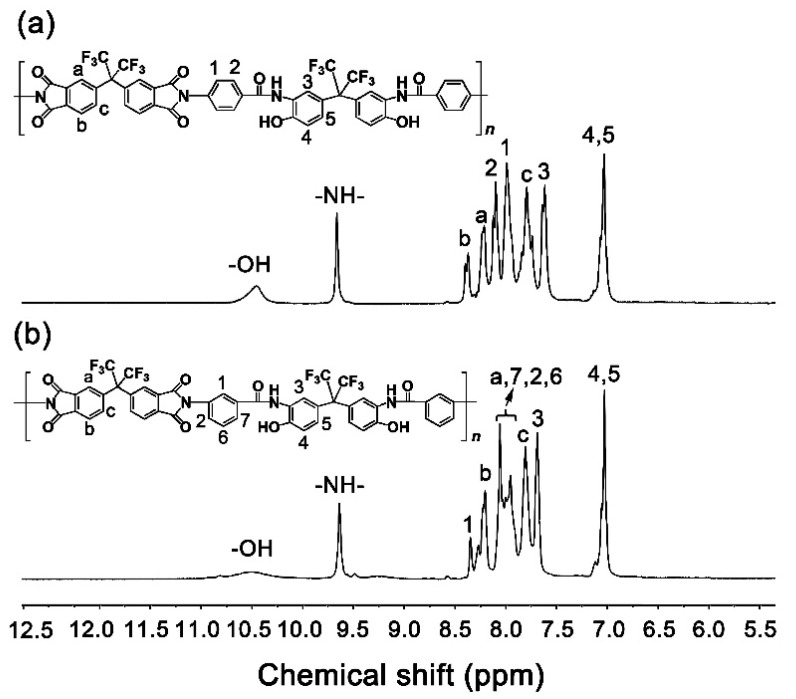
^1^H-NMR spectra of PI NFMs (in DMSO-*d*_6_). (**a**) PI-1; (**b**) PI-2.

Figure 6 shows the ^1^H-NMR spectra of PI NFMs in DMSO-*d*_6_. The protons in the two polymers exhibited similar absorptions due to the analogous chemical environments. The protons in the –OH groups showed absorptions at the furthest downfield (~10.4 ppm) in both of the spectra of the polymers. As regards the protons in the polar –CONH– bonds, they showed absorption second furthest downfield (~9.6 ppm) in the spectra. The protons in the aromatic rings showed absorption in the range of 7.0–8.5 ppm in the spectra. The information revealed by the ^1^H-NMR spectra was consistent with the expected structural features of the PIs.

### 3.3. Electrospun PIBO NFMs Preparation

**Figure 7:**
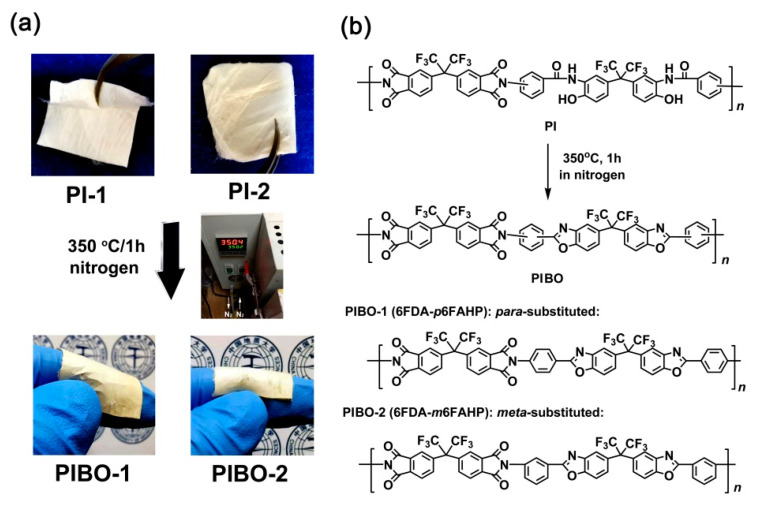
Preparation of PIBO NFMs (**a**) and the corresponding chemical reactions (**b**).

**Figure 8:**
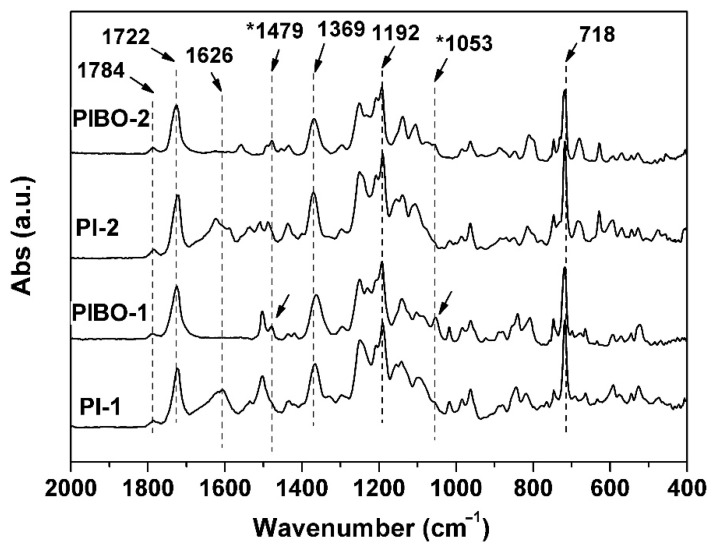
ATR-FTIR spectra of PIBO NFMs.

The PIBO NFMs were prepared by further thermally dehydrated at 350 °C for 1 h in nitrogen, as show in Figure 7a. During the thermal treatment, dehydration reaction occurred in the *ortho*-hydroxyl-substituted benzamide units to form benzoxazole rings according to the chemical process shown in Figure 7b. The successful conversion from the PIs to PIBOs can be confirmed by the ATR-FTIR measurements depicted in Figure 8. It can be clearly observed that the characteristic absorptions of N-H deformation vibrations (1626 cm^−1^) in primary amide (–CONH–) in PI-1 and PI-2 all disappeared in the spectra of corresponding PIBOs. More importantly, the characteristic absorption of benzoxazoles at 1479 cm^−1^ (–C–N– stretching vibrations) and 1053 cm^−1^ (–O–C stretching vibrations) was clearly recorded in the spectra of the PIBOs. Meanwhile, the characteristic absorptions of imide rings (1784 cm^−1^, 1722 cm^−1^, 1369 cm^−1^, and 718 cm^−1^) were maintained in the spectra of PIBOs. The information indicated the successful preparation of the PIBO NFMs.

**Figure 9:**
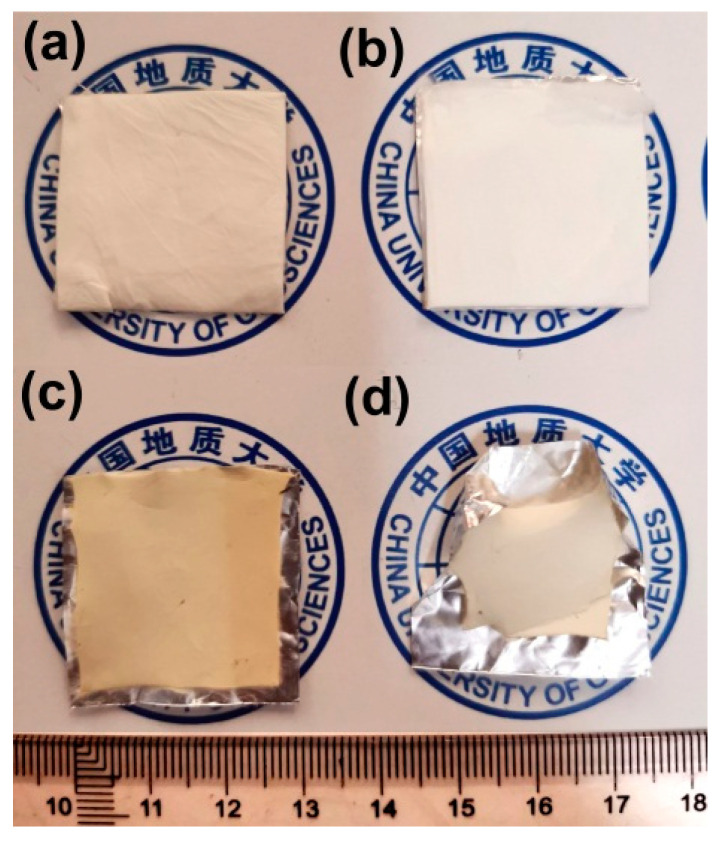
Shrinkage phenomena during the transition from PI NFMs to PIBO NFMs. (**a**) PI-1; (**b**) PI-2; (**c**) PIBO-1; (**d**) PIBO-2.

Water by-products were released off the PI NFMs during the thermal cyclization process, which caused some macroscopic and microscopic changes for the NFMs. Figure 9 shows the macroscopic changes of the NFMs after thermal treatment. Apparently, the high-temperature procedure caused the warpage and shrinkage of the NFMs, especially for PI-2 NFM. The shrinkage is on the one hand due to the release of the water by-products, and on the other hand might be due to the molecular chains movements induced by the glass transition at elevated temperatures, which will be discussed in detail below.

**Figure 10:**
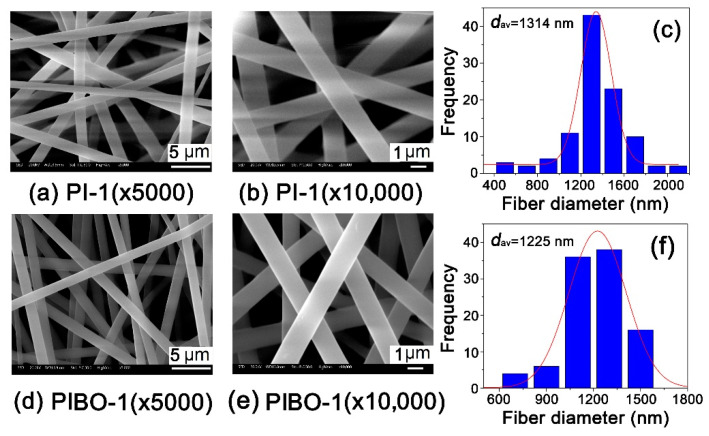
Micro-morphologic scanning electron microscopy (SEM) images and average diameters (*d*_av_) of PI-1 and PIBO-1 NFMs. (**a**) PI-1 (×5000); (**b**) PI-1 (×10,000); (**c**) PI-1 (*d*_av_); (**d**) PIBO-1 (×5000); (**e**) PIBO-1 (×10,000); (**f**) PIBO-1 (*d*_av_).

**Figure 11:**
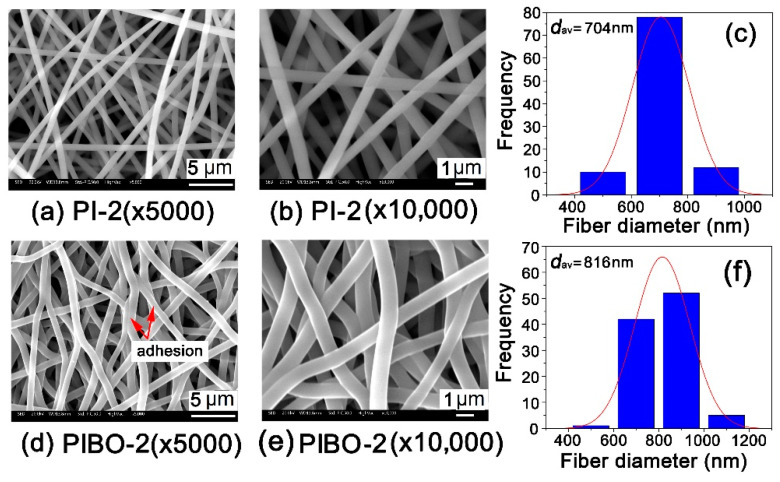
Micro-morphologic SEM images and average diameters (*d*_av_) of PI-2 and PIBO-2 NFMs. (**a**) PI-2 (×5000); (**b**) PI-2 (×10,000); (**c**) PI-2 (*d*_av_); (**d**) PIBO-2 (×5000); (**e**) PIBO-2 (×10,000); (**f**) PIBO-2 (*d*_av_).

As for the microscopic changes, Figure 10 and Figure 11 show the micromorphologic SEM images and average diameters (*d*_av_) of PI and PIBO NFMs, respectively. First, the PI-1 and PI-2 NFMs consisted of unregularly aligned fine fibers with the average diameters (*d*_av_) of 1314 nm for PI-1 and 704 nm for PI-2, indicating the good fiber-forming abilities of the PIs and the reasonable electrospinning conditions. Secondly, the thermal treatment caused a little bit of shrinkage for the NFMs. As shown in Figure 10, for PI-1 and PIBO-1 systems, the *d*_av_ value decreased from 1314 nm to 1225 nm. Nevertheless, the PIBO-1 fibers exhibited fine micromorphology with few defects. However, for the PI-2 and PIBO-2 systems, the high-temperature process caused partial adhesion between the individual fibers, as illustrated in Figure 11. This adhesion led to the increase of *d*_av_ value from 704 nm for PI-2 to 816 nm for PIBO-2. The adhesion in the PIBO fibers might decrease the porosity of the NFMs, which was disadvantageous for the dielectric properties.

**Figure 12:**
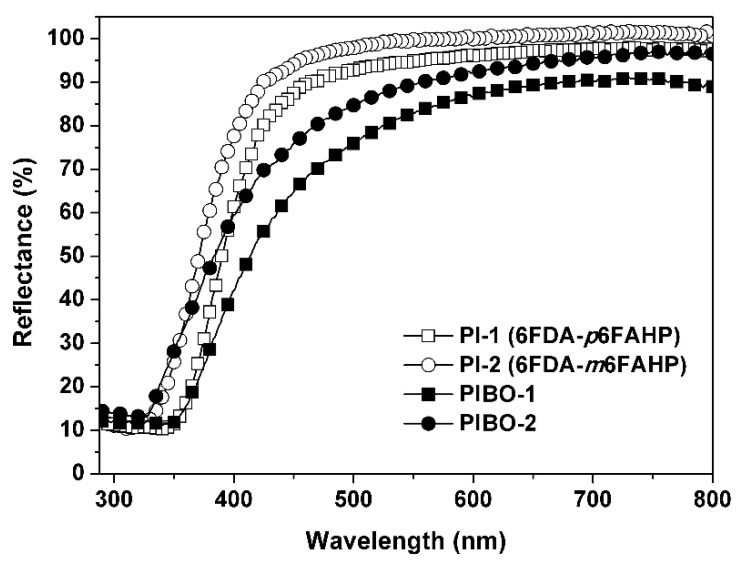
Ultraviolet-visible (UV-Vis) reflectance plots of PI and PIBO NFMs.

**Figure 13:**
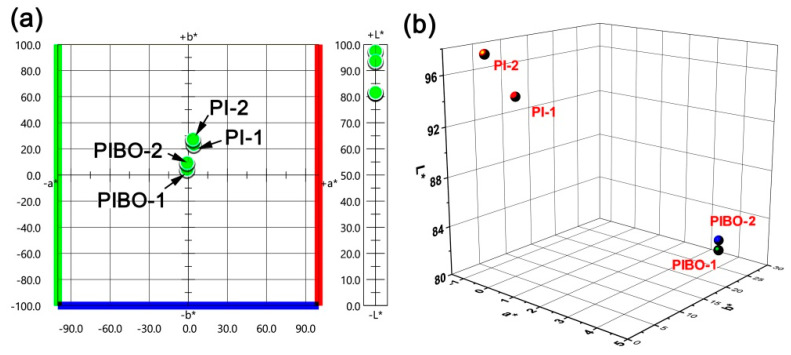
Two-dimensional (2D) (**a**) and 3D (**b**) maps for CIE (Commission Internationale de l´Eclairage) Lab parameters of PI and PIBO NFMs.

In addition, the thermal treatment process also affected the optical properties of the NFMs. Figure 12 and Figure 13 show the UV-Vis reflectance spectra and the CIE Lab optical parameters of the PI and PIBO NFMs, respectively, and the optical data are summarized in Table 2. It can be deduced from Figure 12 that at the wavelength of 457 nm, the PI NFMs showed much higher reflectance (*R*_457_) values than those of PIBO NFMs. For example, PI-1 NFM had the *R*_457_ value of 89.1%, which was 22.1% higher than that of the corresponding PIBO-1. The coloration due to the high-temperature treatment might be the main reason for the low reflectance of the PIBO NFMs. In addition, the PI-2 and PIBO-2 exhibited higher *R*_457_ values than those of the analogous PI-1 and PIBO-1, which was mainly attributed to the *meta*-substituted molecular structures of the former polymers. Finally, all of the PI and PIBO NFMs showed much higher *R*_457_ values than that of the PI-ref NFM (*R*_457_ = 37.3%) derived from PMDA and ODA. This is due to the presence of highly electronegative hexafluoroisopropylidene groups in the PI and PIBO polymers, which efficiently prohibited the charge transfer behaviors from the electron-donating diamine units to the electron-withdrawing dianhydride units [38]. Effects of the high-temperature procedure on the optical features of the polymer NFMs could further be reflected by the CIE Lab measurements, as could be deduced from Figure 13 and Table 2. It could be seen that the high-temperature treatment decreased the lightness (L^*^) and increased the yellow indices (b^*^) of the polymer NFMs. The polymer NFMs showed the whiteness indices (WI) with the decreasing order of PI-2 > PI-1 > PIBO-2> PIBO-1 > PI-ref. This trend is consistent with the change of *R*_457_ values of the polymers.

### 3.4. Thermal Properties

**Figure 14:**
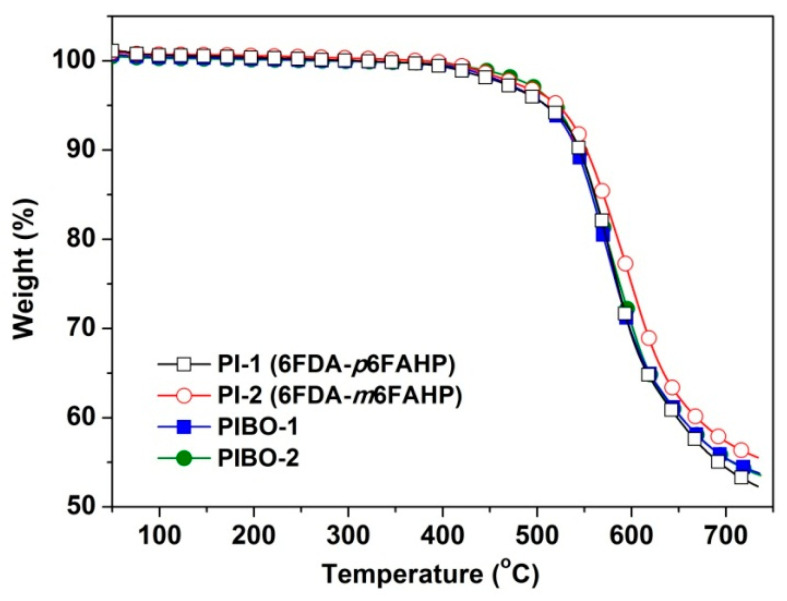
Thermogravimetric analysis (TGA) plots of PI and PIBO NFMs in nitrogen.

The influences of the chemical structures of the polymer NFMs on their thermal properties were studied by TGA and DSC measurements and the data are shown in Table 3. Figure 14 depicts the TGA plots of the polymer NFMs in nitrogen. All the polymers maintained their original weights up to 450 °C. Then, the polymers started decomposing with the increasing temperatures and showed 5% weight loss temperatures (*T*_5%_) in the range of 509–522 °C and residual weight ratios at 700 °C (*R*_w700_) values around 55%. The PI NMF and the corresponding PIBO NMF showed quite similar thermal decomposition behaviours. For example, PI-1 showed a *T*_5%_ value of 510 °C, which is almost the same with that of PIBO (*T*_5%_ = 509 °C). This phenomenon might be due to the similar chemical compositions of the polymers at elevated temperatures. During the measurements, the PI dehydrated first when the temperature was over 350 °C and the corresponding PIBO formed. The tiny weight loss due to the dehydration of PI was not clearly detected by the measurements. This resulted in the similar thermal decomposition behaviours of the polymers. The *meta*-substituted polymers (PI-2 and PIBO-2) showed slightly higher *T*_5%_ values than those of the *para*-substituted counterparts (PI-1 and PIBO-1).

**Figure 15:**
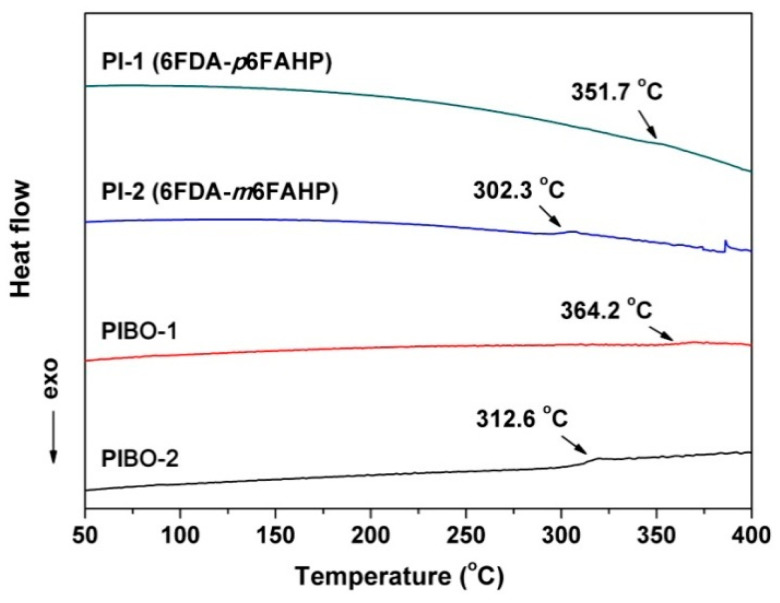
Differential scanning calorimetry (DSC) curves of PI and PIBO NFMs in nitrogen.

Figure 15 shows the DSC plots of the polymer NFMs. The PIBOs showed higher glass transition temperatures (*T*_g_s) than those of the corresponding PIs. For example, PIBO-1 had the *T*_g_ of 364.2 °C, which was 12.5 °C higher than that of PI-1. The rigid benzoxazole rings in PIBOs efficiently prohibited the free motion of the molecular chains at elevated temperatures, resulting in high *T*_g_ values for the polymers. In addition, PI-1 and PIBO-1 with the *para*-substituted molecular structures showed obviously higher *T*_g_ values than those of the analogous *meta*-substituted ones (PI-2 and PIBO-2). For example, PIBO-1 possessed a *T*_g_ value of 364.2 °C, which was 51.6 °C higher than that of PIBO-2. It should be noted that the thermal conversion temperature from PI to PIBO was 350 °C in the current work, which was higher than the *T*_g_ value of PIBO-2 (*T*_g_ = 312.6 °C). Thus, this explains the adhesion among the PIBO-2 fibers shown in Figure 11. As mentioned before, the adhesion interaction in PIBO-2 was disadvantageous for the dielectric properties.

### 3.5. Dielectric Properties

**Figure 16:**
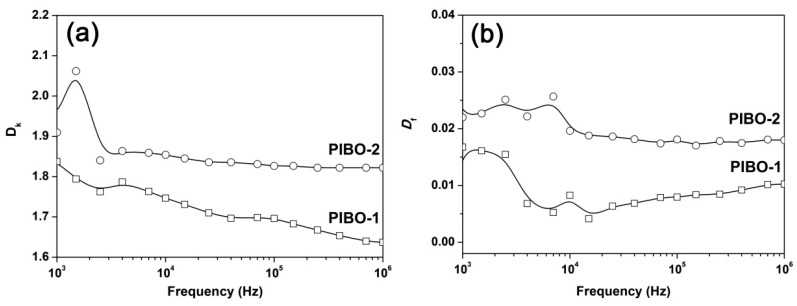
*D*_k_ and *D*_f_ values of PIBO NFMs as a function of frequency. (**a**) *D*_k_ values; (**b**) *D*_f_ values.

The dielectric properties, including dielectric constant (*D*_k_) and dissipation loss factor (*D*_f_) of the PIBO NFMs were measured and the results are shown in Table 3. Figure 16 depicts the *D*_k_ and *D*_f_ values of PIBO NFMs as a function of frequency from 10^3^ to 10^6^ Hz. It has been well-established that the electrospun fibrous membranes usually show much lower *D*_k_ values than those of the free-standing solid films with the same structures, either for common polymers [39], or for PIs [40]. The intrinsically porous structures with filled air (*D*_k_ ≈ 1.0) pores and high surface-to-volume ratio of the fibers in the nano-fibrous membranes are thought to be the main reasons for the low-*D*_k_ features of such materials. Meanwhile, the structural features of the polymer skeletons also contribute to the *D*_k_ values of the electrospun mats. Polymers with low moisture or water (*D*_k_ ≈ 80) absorption, that is, good hydrophobicity, usually provide electrospun mats with lower *D*_k_ values.

In the current work, the PIBO NFMs showed the *D*_k_ value of 1.64 for PIBO-1 and 1.82 for PIBO-2 at the frequency of 1 MHz, respectively (Figure 16a). There is no doubt that for the electrospun nano-porous mats, the effect of porous structure on the dielectric constants is much higher than that of the polymer bulk properties. The solid free-standing fluoro-containing PI or PBO films usually exhibit the *D*_k_ values higher than 2.0 at 1 MHz even if high fluoro contents were introduced, such as the PI (6FDA-12FDA) and PI (6FDA-15FDA) shown in Table 3 [16]. Although the high-fluoro-content PIs exhibited similar thermal stability with the current PI and PBOs, the *D*_k_ values are much higher than those of the NFMs. In addition, the *meta*-substituted PIBO-2 should generally exhibit a lower *D*_k_ value than that of the *para*-substituted PIBO-1 due to the increased free volumes of the molecular chains. However, the partial adhesion among the fibers that occurred inside the PIBO-2 mats decreased their porosity, thus increasing the *D*_k_ values of the mats. Finally, the PIBO NMFs showed *D*_f_ values in the range of 0.010–0.018 at 1 MHz, which was acceptable for their practical application.

## 4. Conclusions

Novel electrospun PIBO nano-fibrous membranes were designed and prepared in the current work. The fabricated PIBO NFMs possesses several structural and compositional features, including high fluoro contents, rigid molecular chain skeleton, and air-filled nano-porous morphology. These features endowed the PIBO NFMs with many desirable properties as high-performance dielectrics for advanced communication devices, such as high thermal stability, low moisture absorption, good dielectric characteristics at high frequency, and so on. PIBO-1 showed the best combined properties, including high-*T*_g_ (364.2 °C), low *D*_k_ (1.64@1 MHz), low *D*_f_ (0.01@1 MHz), and acceptable optical reflectance and whiteness. It can be anticipated that the current PIBO NFMs might be good candidates for microelectronic and communication applications.

## Figures and Tables

**Table 1:** Inherent viscosities, molecular weights, and solubility of PI resins.

PI	[*ƞ*]_inh_^1^ (dL g^−1^)	Molecular Weight ^2^ (×10^4^ g mol^−1^)			Solubility ^3^				
		*M*_n_	*M*_w_	PDI	NMP	DMAc	DMF	CHCl_3_	THF
PI-1	0.78	3.08	5.07	1.65	++	++	++	+−	−
PI-2	0.73	3.01	4.68	1.56	++	++	++	++	−

^1^ Inherent viscosities measured with a PI resin at a concentration of 0.5 g dL^−^^1^ in NMP at 25 °C; ^2^ *M*_n_: number average molecular weight; *M*_w_: weight average molecular weight; PDI: polydispersity index, PDI = *M*_w_/*M*_n_; ^3^ ++: Soluble; +−: partially soluble; −: insoluble; CHCl_3_: chloroform; THF: tetrahydrofuran.

**Table 2:** Optical properties of PI and PIBO NFMs.

PI	*R*_457_^1^ (%)	L* ^2^	a* ^2^	b* ^2^	WI ^3^
PI-1	89.1	93.71	−0.83	9.45	88.6
PI-2	95.3	97.46	−0.86	4.31	94.9
PIBO-1	67.0	81.64	3.62	27.53	66.7
PIBO-2	77.5	81.88	4.21	23.46	70.1
PI-ref	37.3	84.65	1.62	37.96	59.0

^1^*R*_457_: Reflectance at the wavelength of 457 nm; ^2^L*, a*, b*: color parameters calculated according to a CIE Lab equation. L* is the lightness, where 100 means clear and 0 implies black. a*: positive value means red, negative value indicates green. b*: positive value means yellow, negative value indicates blue; ^3^ WI: whiteness.

**Table 3:** Thermal and dielectric properties of PI and PIBO NFMs.

Samples	*T*_5%_^1^ (°C)	*T*_10%_^1^ (°C)	*R*_w700_^1^ (%)	*T*_g_^1^ (°C)	*D*_k_^2^ (1 MHz)	*D*_f_^2^ (1 MHz)
PI-1	510	546	54.4	351.7	ND ^3^	ND
PI-2	522	552	57.3	302.3	ND	ND
PIBO-1	509	542	55.5	364.2	1.64	0.010
PIBO-2	519	545	55.4	312.6	1.82	0.018
6FDA-12FDA ^4^	510	529	54.2	227.5	2.56	ND
6FDA-15FDA ^4^	504	522	43.1	234.2	2.49	ND

^1^*T*_5%_: 5% weight loss temperature; *T*_10%_: weight loss temperature; *R*_w700_: residual weight ratio at 700 °C in nitrogen; *T*_g_: glass transition temperature; ^2^*D*_k_: dielectric constant; *D*_f_: dielectric dissipation factor. ^3^ Not detected. ^4^ PI films with high fluoro contents [16].

## Data Availability

Data is contained within the article.

## References

[1] Jilani S.F., Munoz M.O., Abbasi Q.H., Alomainy A. (2019). Millimeter-wave liquid crystal polymer based conformal antenna array for 5G applications. IEEE Antennas Wirel. Propag. Lett..

[2] Zhou J., Tao Y., Chen X., Chen X., Fang L., Wang Y., Sun J., Fang Q. (2019). Perfluorocyclobutyl-based polymers for functional materials. Mater. Chem. Front..

[3] Martin S.J., Godschalx J.P., Mills M.E., Shaffer II E.O., Townsend P.H. (2000). Development of a low-dielectric-constant polymer for the fabrication of integrated circuit interconnect. Adv. Mater..

[4] Treichel H. (2001). Low dielectric constant materials. J. Electron. Mater..

[5] Volksen W., Miller R.D., Dubois G. (2010). Low dielectric constant materials. Chem. Rev..

[6] Zhao X.Y., Liu H.J. (2010). Review of polymer materials with low dielectric constant. Polym. Int..

[7] Maex K. (2003). Low dielectric constant materials for microelectronics. J. Appl. Phys..

[8] Yao B., Hong W., Chen T., Han Z., Xu X., Hu R., Hao J., Li C., Li H., Perini S.E. (2020). Highly stretchable polymer composite with strain-enhanced electromagnetic interference shielding effectiveness. Adv. Mater..

[9] Wang Z., Zhang M., Han E., Niu H., Wu D. (2020). Structure-property relationship of low dielectric constant polyimide fibers containing fluorine groups. Polymer.

[10] Ma S., Wang S., Jin S., Wang Y., Yao J., Zhao X., Chen C.H. (2020). Construction of high-performance, high-temperature shape memory polyimides bearing pyridine and trifluoromethyl group. Polymer.

[11] Liu T.Q., Zheng F., Ma X., Ding T.M., Chen S., Jiang W., Zhang S.Y., Lu Q. (2020). High heat-resistant polyimide films containing quinoxaline moiety for flexible substrate applications. Polymer.

[12] Lin S., Joo T., Benedetti F.M., Chen L.C., Wu A.X., Rodriguez K.M., Qian Q., Doherty C.M., Smith Z.P. (2021). Free volume manipulation of a 6FDA-HAB polyimide using a solid-state protection/deprotection strategy. Polymer.

[13] Ma P., Dai C., Wang H., Li Z., Liu H., Li W., Yang C. (2019). A review on high temperature resistant polyimide films: Heterocyclic structures and nanocomposites. Compos. Commun..

[14] Chu S., Pan Y., Wang Y., Zhang H., Xiao R., Zou Z. (2020). Polyimide-based photocatalysts: Rational design for energy and environmental applications. J. Mater. Chem. A.

[15] Simpson J.O., St. Clair A.K. (1997). Fundamental insight on developing low dielectric constant polyimides. Thin Solid Films.

[16] Tao L., Yang H., Liu J., Fan L., Yang S. (2009). Synthesis and characterization of highly optical transparent and low dielectric constant fluorinated polyimides. Polymer.

[17] Chern Y.T., Tsai J.Y. (2008). Low dielectric constant and high organosolubility of novel polyimide derived from unsymmetric 1,4-bis(4-aminophenoxy)-2,6-di-tert-butylbenzene. Macromolecules.

[18] Ma S., Wang Y., Min Z., Zhong L. (2013). Nano/mesoporous polymers based low-*k* dielectric materials: A review on methods and advances. Adv. Polym. Technol..

[19] Kourakata Y., Onodera T., Kasai H., Jinnai H., Oikawa H. (2021). Ultra-low dielectric properties of porous polyimide thin films fabricated by using the two kinds of templates with different particle sizes. Polymer.

[20] Kim J., Kwon J., Kim M., Do J., Lee D., Han H. (2016). Low-dielectric-constant polyimide aerogel composite films with low water uptake. Polym. J..

[21] Kohl P.A. (2011). Low-dielectric constant insulators for future integrated circuits and packages. Annu. Rev. Chem. Biomol. Eng..

[22] Tao L., Yang H., Liu J., Fan L., Yang S. (2010). Synthesis of fluorinated polybenzoxazoles with low dielectric constants. J. Polym. Sci. Part A Polym. Chem..

[23] Wang L., Liu C., Shen S., Xu M., Liu X. (2020). Low dielectric constant polymers for high speed communication network. Adv. Ind. Eng. Polym. Res..

[24] Fukukawa K., Shibasaki Y., Ueda M. (2004). A photosensitive semi-alicyclic poly(benzoxazole) with high transparency and low dielectric constant. Macromolecules.

[25] Guo D.D., Jiang J.W., Liu Y.J., Liu X.L., Sheng S.R. (2015). New fluorinated xanthene-containing polybenzoxazoles with low dielectric constants. J. Fluor. Chem..

[26] Zhang H., Jiang S., Duan G., Li J., Liu K., Zhou C., Hou H. (2014). Heat-resistant polybenzoxazole nanofibers made by electrospinning. Eur. Polym. J..

[27] Lee M.J., Kim J.H., Lim H.S., Lee S.Y., Yu H.K., Kim J.H., Lee J.S., Sun Y.K., Guiver M.D., Suh K.D. (2015). Highly lithium-ion conductive battery separators from thermally rearranged polybenzoxazole. Chem. Commun..

[28] Hsu S.L.C., Lin S.S., Wang C. (2008). Preparation of polybenzoxazole fibers via electrospinning and postspun thermal cyclization of polyhydroxyamide. J. Polym. Sci. Part A Polym. Chem..

[29] Park J.H., Ahn B.H. (2016). Synthesis of polyimides derived from 2,2-bis[4-(4-aminobenzoyl)phenoxy]- hexafluoropropane and aromatic dianhydrides. Appl. Chem. Eng..

[30] Hu X., Lee W.H., Zhao J., Kim J.S., Wang Z., Yan J., Zhuang Y., Lee Y.M. (2020). Thermally rearranged polymer membranes containing highly rigid biphenyl ortho-hydroxyl diamine for hydrogen separation. J. Membr. Sci..

[31] Li T., Liu T., Jiao Y., Dong J., Gan F., Zhao X., Zhang Q. (2019). Novel high-performance poly(benzoxazole-co-imide) resins with low dielectric constants and superior thermal stability derived from thermal rearrangement of ortho-hydroxy polyimide oligomers. Chem. Eng. J..

[32] Zhang K., Han L., Froimowicz P., Ishida H. (2017). A smart latent catalyst containing o-trifluoroacetamide functional benzoxazine: Precursors for low temperature formation of very high performance polybenzoxazole with low dielectric constant and high thermal stability. Macromolecules.

[33] Guo C.Y., Wang Q.W., Liu J.G., Qi L., Huangfu M.G., Wu X., Zhang Y., Zhang X.M. (2019). Electrospun polyimide ultrafine non-woven fabrics with high whiteness and good thermal stability from organo-soluble semi-alicyclic polyimides: Preparation and properties. Express Polym. Lett..

[34] Topuz F., Abdulhamid M.A., Nunes S.P., Szekely G. (2020). Hierarchically porous electrospun nanofibrous mats produced from intrinsically microporous fluorinated polyimide for the removal of oils and non-polar solvents. Environ. Sci. Nano.

[35] Jiang S., Hou H., Agarwal S., Greiner A. (2016). Polyimide nanofibers by “Green” electrospinning via aqueous solution for filtration applications. ACS Sustain. Chem. Eng..

[36] Topuz F., Abdulhamid M.A., Holtzl T., Szekely G. (2021). Nanofiber engineering of microporous polyimides through electrospinning: Influence of electrospinning parameters and salt addition. Mater. Design.

[37] He Y., Han D., Chen J., Ding Y., Jiang S., Hu C., Chen S., Hou H. (2014). Highly strong and highly tough electrospun polyimide/polyimide composite nanofibers from binary blend of polyamic acids. RSC Adv..

[38] Guo C.Y., Liu J.G., Yin L.M., Huangfu M.G., Zhang Y., Wu X., Zhang X.M. (2018). Preparation and characterization of electrospun polyimide microfibrous mats with high whiteness and high thermal stability from organo-soluble polyimides containing rigid-rod moieties. Fibers Polym..

[39] Chen F., Bera D., Banerjee S., Agarwal S. (2012). Low dielectric constant polyimide nanomats by electrospinning. Polym. Adv. Technol..

[40] Sharma B., Verma R., Baur C., Bykova J., Mabry J.M., Smith D.W. (2013). Ultra-low dielectric, self-cleansing and highly oleophobic POSS-PFCP aryl ether polymer composites. J. Mater. Chem. C.

